# Quantifying economic vulnerabilities induced by interdependent networks

**DOI:** 10.1371/journal.pone.0306893

**Published:** 2024-07-11

**Authors:** Shokoufeh Pourshahabi, Shade T. Shutters, Rachata Muneepeerakul

**Affiliations:** 1 Department of Agricultural and Biological Engineering, University of Florida, Gainesville, FL, United States of America; 2 School of Complex Adaptive Systems, Arizona State University, Tempe, AZ, United States of America; University of Foggia: Universita degli Studi di Foggia, ITALY

## Abstract

Under economic globalization, countries are linked through trade and investments. This economic interdependence creates vulnerabilities. The indirect vulnerability induced by interdependent networks of trade and investments can put a country’s economy at risk, but this risk has yet to be systematically quantified and investigated. In this paper, we developed the novel Potential Indirect Vulnerability Index (PIVI) to capture how interdependencies between networks of trade and foreign direct investment (FDI) may induce economic vulnerabilities. The model consisted of three main components: a target country (the importer of goods), an investing country (the exporter of FDI), and the intermediary countries that export commodities to the target country and receive FDI from the investing country, serving as conduits of the vulnerabilities caused indirectly by the investing country. The PIVI quantifies the indirect vulnerabilities based on the product of two fractions: 1) the dependency of the target country on commodities from each intermediary country; and 2) the dependency of each intermediary country on FDI from the investing country. We demonstrated the utility of PIVI by examining the US economy’s vulnerability to China using 2019 trade and FDI data. Several Asian countries and a mix of agricultural products and raw materials were identified as conduits through which China could potentially influence the US economy. Vietnam was a sizeable risk because, while it has been a primary source of many US imports, it also received about 30% of its FDI from China. The US policy makers might opt to increase diversity in trade partners or to promote investment in countries such as Vietnam. We also applied the PIVI analysis to critical minerals, identifying cobalt, tungsten, and copper as the most vulnerability-inducing among them. PIVI is a flexible metric than can be aggregated and modified to provide a more nuanced and focused assessment of an economy’s vulnerability.

## Introduction

Economic globalization has fostered a remarkable degree of interconnectedness among countries worldwide, giving rise to intricate and complex interdependencies within trade systems [1–4]. Economic interdependence through trade was long viewed as desirable due to the belief that economically interdependent countries are less likely to engage in conflict with one another [5, 6].

However, interdependence can also create risks. Many studies have shown that as interconnectedness within a networked system increases, the system can become more vulnerable to cascading failures and disruptions with far-reaching consequences [7–12]. These vulnerabilities extend to economic and trade networks [13–16]. Some of these risks, can be readily identified (although not always easy to manage), such as low diversity of trade partners, partners too far away, partners in politically unstable areas, partners in environmentally unstable areas, etc. [17]. For instance, when drought struck wheat-growing regions of China in 2011, the shock cascaded through the global food trade network contributing to distant impacts such as the fall of Egypt’s government the following year [18]. In this example, China’s increased import of wheat directly affected the global wheat trade network and indirectly impacted wheat-importing countries like Egypt. These direct and indirect effects were relatively easy to identify because they occurred within a trade network of a single commodity.

However, there also exist indirect risks that arise not simply through trade relationships but through linkages *between* trade networks and other networks [19–21]. These indirect risks operate indirectly through intermediary countries linking two different networks, thereby making them less apparent. Indeed, in today’s intricately connected world, many risks associated with network connectedness arise not only from interconnections within individual networks, but also from interdependencies *between* networks [22, 23]. For example, though Italy’s internet and power networks were designed to be robust in isolation, the networks were interdependent such that the collapse of a small part of the electrical grid in 2010 led to cascading failures in both networks, resulting in national-scale blackouts of both the internet and power grid [24].

Many scholars have developed network metrics to assess vulnerabilities in networked systems such as international trade network [25, 26], cobalt trade network [27], internet infrastructure [28], and transportation network [29–31]. Li et al. (2014), for instance, used Wassily Leontief input-output model to quantify vulnerabilities in the interdependent financial network. They determined critical industries and countries based on potential global damage resulting from their failures. Their research emphasized China’s growing economic dominance, within the global economy [19]. All of these studies have focused on the vulnerabilities within individual networks: measuring vulnerabilities in interdependent multi-layered networks has, however, been understudied. Thus, the primary motivation of this study is to quantify less apparent but potential vulnerabilities hidden in the complexities of interdependent networks.

As interdependent networks as a whole can be vulnerable to a disruption at a small part of one of the networks, it is critical to have better understanding of vulnerability of a node embedded in such interdependent networks. Here we focus on economic vulnerability of a country’s economy embedded in the interconnected world of commerce. Specifically, we analyze how linkages between networks of commodity trade flows and networks of investment flows can induce vulnerabilities in a national economy that are not obvious when examining only one of the networks in isolation. Consider the following scenario. Country A imports most of its critical goods from country B. Country B depends heavily on country C for its foreign investments. There exists political tension between countries A and C. Country C can be said to have the potential to disrupt country A’s economy by pressuring country B to restrict the flows of the critical goods into country A. Given the interconnectedness among countries brought about by economic globalization and the increasing complexity of global geopolitics, it is not difficult to replace A, B, and C with real countries. Yet, to our knowledge, there is no measure to systematically and quantitatively capture such indirect vulnerability. The main contribution of this research is to address this knowledge gap. Our specific objectives are to:

1. Develop a new index to quantify the indirect vulnerability induced by economic interdependence between networks of commodity trade flows and foreign direct investment flows;
2. Apply the index to a case study to determine indirect vulnerability of a target country (US) against an investing country (China) through intermediary country-commodity pairs to showcase the index’s usefulness;
3. Demonstrate how the results can be aggregated to identify commodities and intermediary countries that serve as conduits of such vulnerability; and
4. Determine the indirect vulnerability of critical minerals identified by RAND Corporation [32] as an example of an application of a specific sector.

The insights derived from this research can be useful for policymakers to effectively anticipate the security implications of economic and trade policies, thereby contributing to the principles of fiscal federalism for addressing economic vulnerabilities and strengthening the resilience of the economy by leveraging the advantages of decentralization, resource allocation, innovation and competition (see, e.g., Ref. [33]).

The paper is structured as follows: In the Methods section, we described Potential Indirect Vulnerability Index, case study, and data. Following this, we presented results and discussion, starting with the fundamental units, namely country-commodity pairs, that induce indirect vulnerability of the US economy to China, followed by aggregating the index to determine countries that are conduits for indirect US economic vulnerability to China and commodities that are conduits for indirect US economic vulnerability to China, as well as sector-specific vulnerability, using critical minerals as an example. We then suggested future work, and finally concluded with a summary of our key findings.

## Methods

### Potential indirect vulnerability index

**Fig 1:**
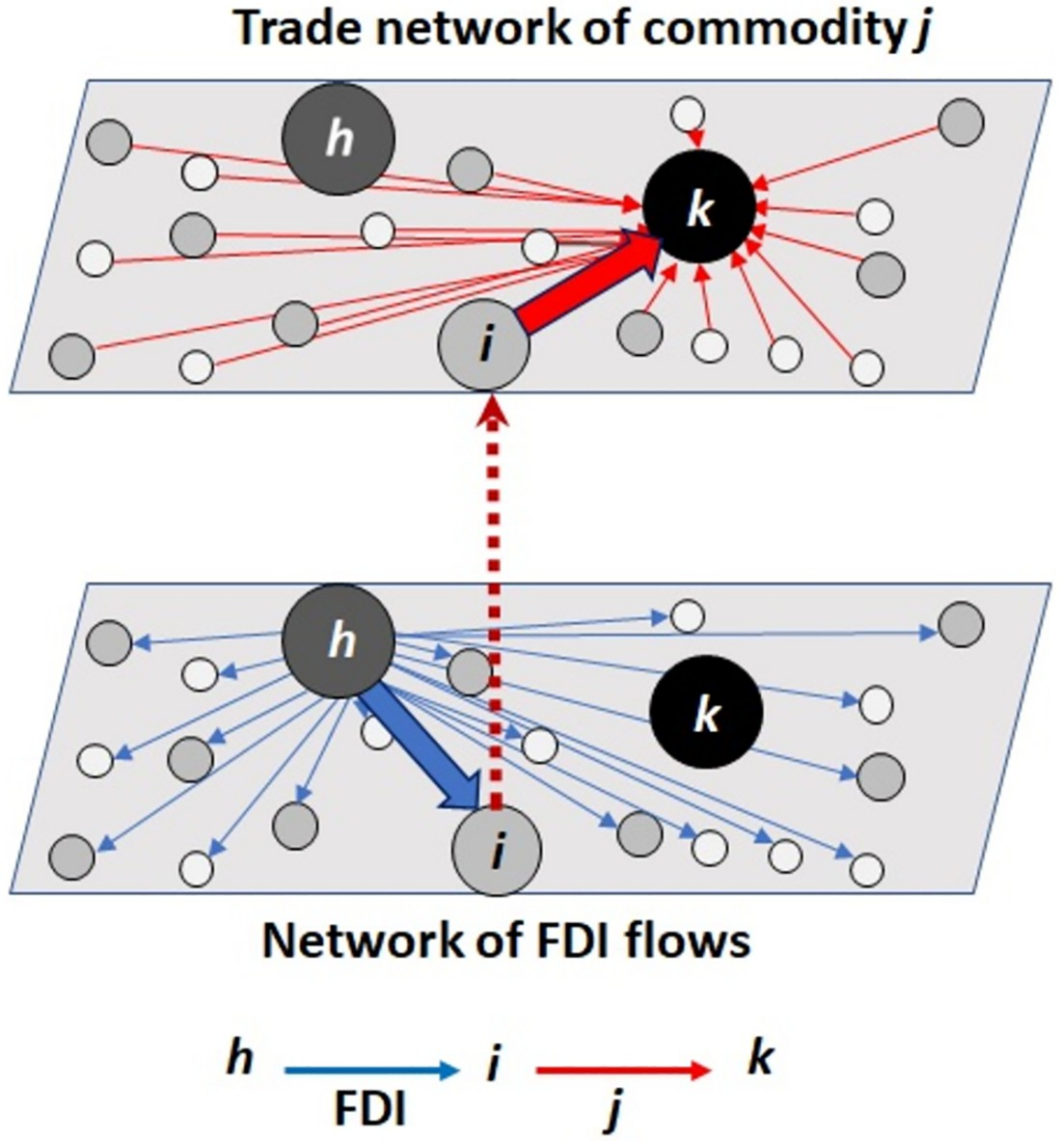
How indirect vulnerabilities arise through linked networks. When a substantial portion of imports to country *k* come from country *i* (top network), and country *i* receives a large portion of its incoming foreign investment from country *h* (bottom network), it creates the possibility for country *h* to exert coercive power over country *k* supply chains. Such cross-linkages between networks give rise to indirect vulnerabilities of country *k*’s economy.

If the country *i* is dependent on country *h* through the inflow of foreign direct investment (FDI) and country *k* is dependent on the country *i* through import of the commodity *j*, this establishes a pathway for potential vulnerability: country *h* may exert its financial influence on country *i* to restrict the flow of commodity *j* to country *k* (Fig 1). To quantify this phenomenon, we define the Potential Indirect Vulnerability Index (PIVI) as follows:

$$\begin{array}{c} {PIVI}_{i,j}^{\left(h,k\right)}=\text{log}\;[N^{2}(\frac{{FDI}_{i}^{\left(h\right)}}{\sum_{n=1}^{N}{FDI}_{i}^{\left(n\right)}})(\frac{{Im}_{i,j}^{\left(k\right)}-{Ex}_{i,j}^{\left(k\right)}}{\sum_{n=1}^{N}({Im}_{n,j}^{\left(k\right)}+{Ex}_{n,j}^{\left(k\right)})})] \end{array}$$ (1)

where

${PIVI}_{i,j}^{\left(h,k\right)}$ : The potential indirect vulnerability index of country *k* against country *h* based on the trade of commodity *j* through country *i*;

${FDI}_{i}^{\left(h\right)}$ : The inflow of FDI from country *h* to country *i*;

${Im}_{i,j}^{\left(k\right)}$ : The volume of import of commodity *j* from country *i* to country *k*;

${Ex}_{i,j}^{\left(k\right)}$ : The volume of export of commodity *j* to country *i* from country *k*;

*N*: Total number of potential partner countries either for commodity or FDI flows (*N* = 222 in 2019).

The first fraction in Eq 1 represents the dependency of country *i* on country *h* through the fraction of its incoming FDI that comes from country *h*. The second fraction represents the dependency of country *k* on the country *i* for commodity *j* through the net import of commodity *j* from country *i*. Put together, this means that if the country *i* is heavily dependent on country *h* for foreign investments and country *k* is heavily dependent on the country *i* for the import of the commodity *j*, ${PIVI}_{i,j}^{\left(h,k\right)}$ would be high. ${PIVI}_{i,j}^{\left(h,k\right)}$ is defined only when there is a positive net import of commodity *j* from country *i* to country *k*. If export exceeds import, we assume that such a commodity does not contribute to the indirect vulnerability. The *N*^2^ can be thought of a scaling factor reflecting the size of the system as well as the potential opportunities to diversify one’s interdependence. Finally, the product of terms in the square brackets in Eq 1 varied across several orders of magnitude; the log transformation was applied so that the PIVI values were easier to compare and discuss.

Note that ${PIVI}_{i,j}^{\left(h,k\right)}=0$ corresponds to the situation in which all countries have the inflow of FDI equally from all *N* countries $\left({FDI}_{i}^{\left(h\right)}/\sum_{n=1}^{N}{FDI}_{i}^{\left(n\right)}=1/N\right)$ and country *k* only imports—with no export—commodity *j* from all *N* partner countries equally $(\left({Im}_{n,j}^{\left(k\right)}-{Ex}_{n,j}^{\left(k\right)}\right)/\left(\sum_{n=1}^{N}\left({Im}_{n,j}^{\left(k\right)}+{Ex}_{n,j}^{\left(k\right)}\right)={Im}_{n,j}^{\left(k\right)}/\sum_{n=1}^{N}{Im}_{n,j}^{\left(k\right)}=1/N\right)$. That is, ${PIVI}_{i,j}^{\left(h,k\right)}=\text{log}[N^{2}\frac{1}{N}\frac{1}{N}]=0$. At the other extreme, when country *k* relies *solely* on country *i* for commodity *j* and country *i* relies *solely* on country *h* for foreign investments, both fractions in Eq 1 are one, and ${PIVI}_{i,j}^{\left(h,k\right)}=\text{log}\;N^{2}$ (for *N* = 222, this is equal to 4.7). These values can be used as benchmarks when considering PIVI values.

We made the following assumptions in developing the PIVI index:

1. We assumed that negative or zero net imports do not contribute to the indirect vulnerability. As an example, the US has imported Lard stearin/lard oil (1503) from Australia (6,045 $US) and exported this commodity to Argentina (30,967 $US), Canada (56,508 $US), China (47,664 $US), Germany (9,936 $US), India (17,234 $US), Japan (36,960 $US), Mexico (2,940,859 $US), Nigeria (1,300,330 $US), Thailand (10,080 $US), and Trinidad and Tobago (45,864 $US) in 2019. Thus, in this analysis, Lard stearin/lard oil (1503) does not affect the indirect vulnerability of the US economy;
2. We assumed that countries that did not receive FDI from country *h* do not contribute to the indirect vulnerability through country *h*; and
3. We did not consider the net inflow of FDI because we cannot obtain the net inflow of FDI by simply subtracting (inflow—outflow). The inflow of FDI reflects investment in different parts of a country’s economy such as manufacturing, industrial activities, infrastructure, finance, healthcare, education, and technology transfer. They cannot be subtracted from each other in the same way as import and export of commodity *j* from country *i*.

### Case study: Indirect vulnerability of the United States to China

As described above, PIVI can be used to determine indirect vulnerability of any country *k* against country *h* through any country-commodity pair *i*-*j*. To this end, it is instructive to apply the PIVI analysis to a specific pair as a case study. In the followings, we will focus on the indirect vulnerability of the US to China, two of the world’s largest economies and most important geopolitical powers (that is, *h* = China and *k* = the US). Below, we provided some brief narrative of their economic interdependence.

In 2022, the US imported approximately $3.31 trillion in goods from other countries and exported about $2.1 trillion [34], highlighting the substantial role of international trade in the US economy. The volume of trade between the United States and China has experienced a remarkable increase since China’s economic reforms and trade liberalization in the late 1970s, rising from $2 billion in 1979 to $636 billion in 2017. This growth has made China an essential and integral part of US global supply chains [35].

At the same time, China’s increasing capabilities present challenges to the US [36]. The US Secretary of Defense recognizes that engaging in long-term strategic competitions with China and Russia is of utmost importance [37]. Such engagement necessitates heightened and sustained investments due to the significant threats they pose to both the current security and prosperity of the US, with the potential for these threats to further escalate in the future.

According to the World Investment Report [38], over the past five years, there has been a notable rise in the volume of projects initiated by Chinese multinational enterprises (MNEs) in developing economies. These endeavors predominantly target the development of transport infrastructure and power generation, extending beyond Asian nations to encompass Africa, Latin America, and the Caribbean. Despite experiencing a marginal three percent decline, China’s outward FDI remained robust at $133 billion, solidifying its position as the world’s leading investor [39].

China actively invests in unstable countries, and those countries are dependent on China for FDI. Global FDI flows to Ethiopia increased from $2.4 billion in 2020 to $4.3 billion in 2021, as China’s investments tripled in 2021. The majority of FDI directed towards landlocked developing countries (LLDCs) primarily originates from a limited number of key investor nations. Among them, China emerges as the predominant investor in LLDCs, with a significant contribution of $20 billion. Notably, China’s investment in Kazakhstan alone amounted to $6 billion, further highlighting its substantial presence and influence in LLDCs [40].

China not only focuses strategically on certain regions, but also on specific materials. China has strategically utilized global interdependence as a tool in the lithium market and Li-ion battery manufacturing. This has enabled China to emerge as the leading producer of these batteries, exercising control over a substantial majority—more than 70 percent—of the global production [41].

All in all, China has continued to hold the top position as the leading contributor to global foreign direct investment [40]. As China continues to assert its influence and shape the global market, this will continue to have implications for industries, economies, and international relations. All these developments, along with the political tension between the US and China, warranted our choice of applying the PIVI analysis to determine the indirect vulnerability of the US against China as a case study.

### Data

#### Trade data

We use the data of bilateral commodity trade flows from the United Nations Comtrade database (http://comtrade.un.org) to obtain the import/export values of the US with partner countries. The Comtrade dataset contains yearly records of the directional flow of goods between countries since 1962. To avoid the influence of the global pandemic caused by the coronavirus, we rely on the most recent pre-pandemic data available, which is from 2019. By using this information, we aim to minimize any potential imbalances or distortions that might have arisen due to the impact of the global pandemic caused by the coronavirus.

We filter out specific records from our dataset to ensure data integrity and accuracy. Firstly, we exclude any records that have a null trading partner or ambiguous trade partners, which does not provide a clear indication of the actual trading entity. Moreover, we discard records with negative trade values and data related to re-exports and re-imports, focusing solely on the direct exchange of goods and services between countries [2]. By applying these exclusions, we aim to maintain the reliability and validity of our data for comprehensive analysis.

In situations where there is a flow of commodities among the US and the partner country, both countries typically report the value of this trade to the United Nations. However, the reported values are not precisely identical. In such cases, we used the average value of the quantities reported by both trading partners to determine the value of the trade among them. Alternatively, if one country reports a value while the partner country does not, we rely on the value submitted by the reporting country [2]. This methodology ensures that we account for any discrepancies and inconsistencies in the reported values, allowing us to obtain a more accurate estimation of the actual trade flow between the two countries.

The Harmonized System (HS) consists of around 5,300 product descriptions, organized into 97 chapters. The six-digit code in the HS can be divided into three parts. The first two digits indicate the chapter in which the goods are classified. For example, HS-01 corresponds to Animals. The next two digits specify groupings within that chapter. For instance, HS-0103 represents Swine. The following two digits provide even more specific details. For example, HS-010391 and HS-010392 refer to Swine (weighing less than 50 kg) and Swine (weighing 50 kg or more), respectively.

Using 6-digit commodity codes may lead to misleading conclusions when we want to calculate the percent of the import of each commodity to the US. If the US imports $X of HS-010391 from one country and $2X of HS-010392 from two other countries ($X from each country), the percent of import of HS-010391 will be 100%, while the percent of the import of HS-010392 for each country will be 50%. However, both HS-010391 and HS-010392 refer to Swine but with different weights. Therefore, we consider 4-digit commodity codes HS-0103 which represents Swine. We believe that the 4-digit level strikes the right balance between commodity specificity and capturing potential vulnerability. Therefore, in this study, we investigate 1,220 products instead of all 5,300 detailed codes, considering 4-digit commodity codes.

#### FDI data

Bilateral flows of Foreign Direct Investment (FDI) is published annually by the United Nations Conference on Trade and Development [38]. We use this data to quantify each country’s financial dependency on China. To ensure data integrity and accuracy, we eliminate any records with null trading partners or ambiguous trade partners, as they do not provide clear indications of the actual trading entities involved. Additionally, we exclude records with negative inflows/outflows of FDI. Negative inflows of FDI can transpire when foreign companies opt to withdraw their investments from a country due to various factors such as economic instability. For more details about the factors that can lead to negative inflows/outflows of FDI, one may refer to World Investment Report [38]. Our analysis is based on the most recent pre-pandemic data, which is from 2019.

## Results and discussion

**Fig 2:**
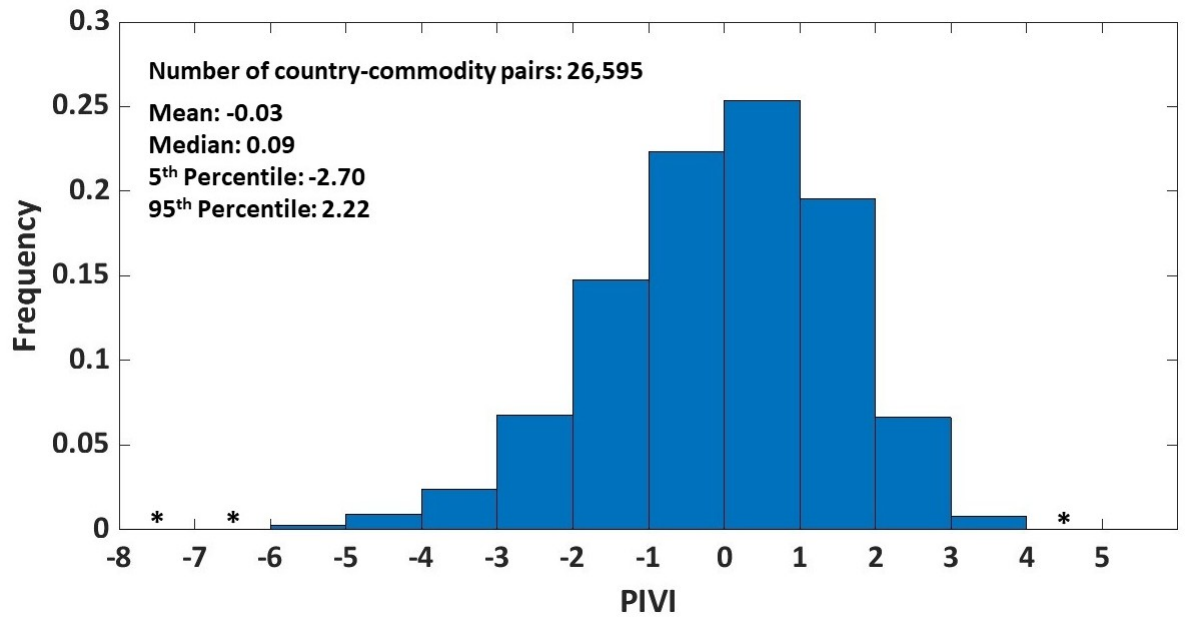
Frequency distribution of PIVI for country-commodity pairs between the US and China in 2019. The frequencies of the bins with * are very small, but not zeros: there are 2, 13, and 3 PIVI values in the [-8,-7), [-7,-6), and [4,5) bins, respectively.

**Fig 3:**
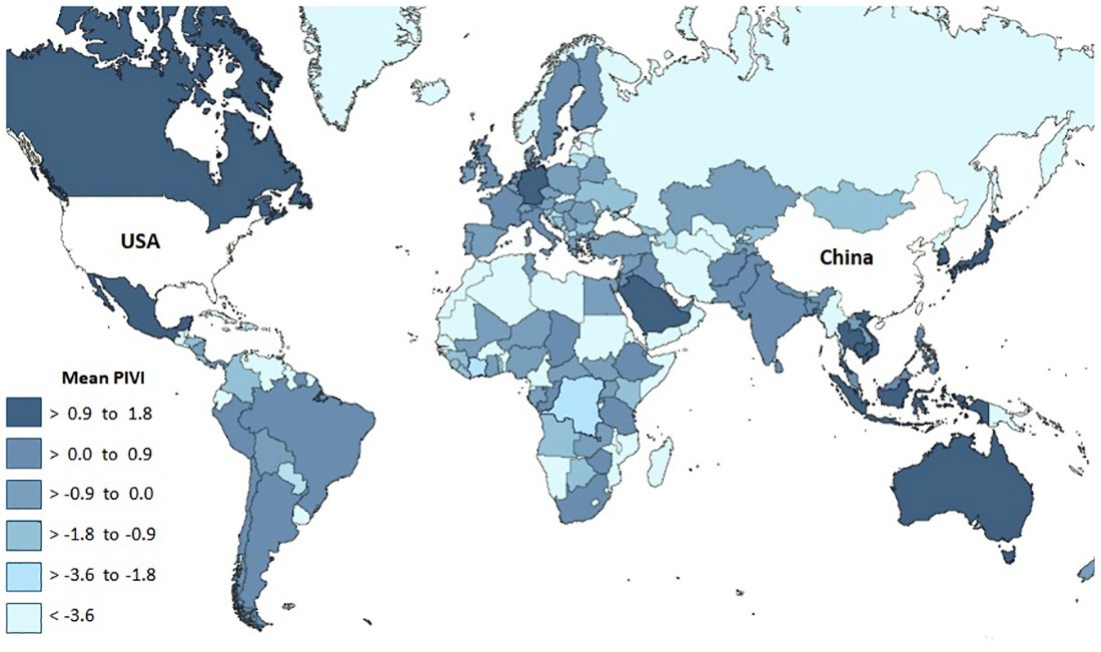
Global map of the degree at which each country was a conduit for indirect US economic vulnerability to China in 2019. The map shows the average PIVI of country *i*, defined as $\left(1/J\right)\sum_{j=1}^{J}{PIVI}_{i,j}^{\left(\text{China},\text{US}\right)}$ where *J* is the number of commodities with net export from country *i* to the US.

We now report the results from the PIVI analysis based on the 2019 trade and FDI data with the US as the target country (the importer of goods) and China as the investing country (the exporter of FDI); that is, *h* and *k* in Eq 1 are fixed as China and the US, respectively (Table 1 and Fig 2). Each PIVI corresponds to a country-commodity (*i*, *j*) pathway (FDI from China to country *i*, which exports commodity *j* to the US). These PIVIs can be aggregated in several ways: they can be aggregated to highlight countries (Table 2 and Fig 3) or commodities (Table 3) that induce such indirect vulnerability or focus on a subset of commodities related to a given sector of an economy (e.g., Table 4). These different aggregated PIVIs provide complementary insights into the indirect vulnerability of an economy.

**Table 1:** Top 10 country-commodity pairs that induce indirect vulnerability of the US to China in 2019. The numbers in parentheses are the 4 digit-level codes of the commodities.

Country-commodity pair	FDI from China %	Net import to USA %	PIVI
Laos–Ores and concentrates (2617)	79.3	40.9	4.20
Bangladesh–Yarn (5307)	31.1	84.3	4.11
Vietnam–Nuts, edible; coconuts, cashew (0801)	29.2	71.9	4.02
Jordan–Garments (6114)	55.0	31.8	3.94
Gabon–Manganese ores and concentrates (2602)	20.1	75.5	3.87
Hong Kong–Clock cases (9112)	62.0	23.4	3.85
Sri Lanka–Retreaded or used pneumatic tyres (4012)	56.5	24.2	3.83
Vietnam–Textile fabrics (5902)	29.2	43.6	3.80
Sri Lanka–Coconut, abaca (5305)	56.5	21.1	3.77
Hong Kong–Cadmium (8107)	62.0	18.7	3.76

**Table 2:** Top 10 countries that potentially induce indirect vulnerability of the US economy to China in 2019 based on their average PIVIs across all commodities.

Rank	Country	Mean PIVI*	FDI from China%	Number of commodities imported to USA	Total import to USA ($billions US)
1	Vietnam	1.80	29.2	526	63.5
2	Hong Kong**	1.80	62.0	376	12.8
3	Canada	1.48	1.3	350	236.0
4	Thailand	1.32	13.4	553	29.4
5	Rep. of Korea	1.29	6.5	512	69.2
6	Saudi Arabia	1.26	30.4	55	13.5
7	Japan	1.22	3.0	565	123.0
8	Germany	1.13	2.6	820	117.0
9	Indonesia	1.04	7.8	438	19.0
10	Cambodia	0.96	37.0	384	5.0

* Here, the mean PIVI of country *i* is defined as $\left(1/J\right)\sum_{j=1}^{J}{PIVI}_{i,j}^{\left(\text{China},\text{US}\right)}$ where *J* is the number of commodities with net export from country *i* to the US.

** Hong Kong is a region in China, but the United Nations classified it with a different code.

**Table 3:** Top 10 commodities that potentially induce indirect vulnerability of the US economy to China in 2019 based on their average PIVI across all countries.

Rank	Commodity code and name	Mean PIVI*	Total import to USA from all countries ($millions US)	Number of partner countries & country with max PIVI
1	0103—Swine	2.78	313.5	1 (Canada)
2	1203—Copra	2.77	0.2	3 (Hong Kong)
3	2716—Electrical energy	2.61	1935.2	2 (Canada)
4	0205—Meat of horses	1.86	2.5	3 (Canada)
5	2705—Coal gas	1.85	0.9	4 (Rep. of Korea)
6	8107—Cadmium	1.85	2.3	12 (Hong Kong)
7	2605—Cobalt ores	1.71	2.5	6 (South Africa)
8	2822—Cobalt oxides/hydroxides	1.67	46.4	16 (Finland)
9	5110—Yarn	1.60	0.1	12 (Nepal)
10	2528—Natural borates	1.43	28.0	13 (Bolivia)

* Here, the mean PIVI of commodity *j* is defined as $\left(1/I\right)\sum_{i=1}^{I}{PIVI}_{i,j}^{\left(\text{China},\text{US}\right)}$ where *I* is the number of intermediary countries with net export of commodity *j* to the US.

**Table 4:** Average PIVI of 19 minerals identified as critical to US manufacturing security by the RAND Corporation report.

Rank	Critical mineral (RAND report*)	HS Commodity code—name	Mean PIVI**
1	Cobalt	2605—Cobalt ores	1.71
2	Tungsten	2611—Tungsten ores	1.30
3	Copper	7405—Copper; master alloys	1.18
4	Niobium	2615—Niobium and vanadium	0.97
5	Vanadium		
6	Antimony	8110—Antimony	0.92
7	Graphite	2504—Graphite; natural	0.66
8	Chromium	2610—Chromium ores	0.64
9	Magnesite	2519—Magnesium carbonate/magnesite	0.50
10	Lithium	2825—Hydrazine***	0.47
11	Magnesium	8104—Magnesium	0.27
12	Rare Earths	2805—Rare-earth metals,	0.26
13	Fluorspar	2529—Feldspar; leucite; nepheline; fluorspar	0.04
14	Germanium	8112—Germanium, gallium, indium, and rhenium	-0.26
15	Gallium		
16	Indium		
17	Rhenium		
18	Platinum Group	7110—Platinum	-1.19
19	Barites	****	****

* Refs [32, 46]

** Here, the mean PIVI of critical mineral *c* is defined as $\left(1/I\right)\sum_{i=1}^{I}{PIVI}_{i,c}^{\left(\text{China},\text{US}\right)}$ where *I* is the number of intermediary countries with net export of mineral *c* to the US.

*** This 4-digit commodity code includes HS-282520, which corresponds to Lithium oxide and hydroxide.

**** This mineral was not imported to the US in 2019.

### Country-commodity pairs that induce indirect vulnerability of the US economy to China

For the 2019 datasets, there are a total of 26,595 country-commodity (*i*, *j*) pairs between China and the US; their PIVIs ranged from -7.6 (least vulnerability) to 4.2 (greatest vulnerability) (Fig 2). Most—about 52%—of the country-commodity pairs have positive PIVI values. Among the top 10 country-commodity pairs, all except Gabon are located in Asia (Table 1). This geographical proximity highlights the interconnectedness of nations within Asia and China’s growing influence in the region. Vietnam, Hong Kong, and Sri Lanka have appeared in the top 10 country-commodity pairs more than once, highlighting their contribution to the indirect vulnerability. The commodities in these top 10 country-commodity pairs were a mix of raw materials, agricultural products, and non-hi-tech manufactured goods, representing a diverse range of industries such as textiles, agriculture, and mining.

### Countries that are conduits for indirect US economic vulnerability to China

To identify countries that potentially induce indirect vulnerability of the US economy to China, we took the average across commodities of each intermediary country’s PIVIs; that is, for country *i*, we calculated $\left(1/J\right)\sum_{j=1}^{J}{PIVI}_{i,j}^{\left(\text{China},\text{US}\right)}$, where *J* is the number of commodities with net export from country *i* to the US. Among the top 10 countries, seven were in Asia and five received more than 10% of their FDI from China (Table 2). At the top of the list was Vietnam, whose almost 30% of its FDI comes from China. The United States imported 526 commodities out of 1,220 4-digit products from Vietnam in 2019, excluding cases where the value of commodities exported from the US exceeds the value of commodities imported to the US. Vietnam, Canada, and Japan are engaged in the Trans-Pacific Partnership (TPP) agreement [42], which managed to proceed despite the withdrawal of the US [43].

One potential way to mitigate such indirect vulnerability is to establish a trade agreement with the Association of Southeast Asian Nations (ASEAN) and the Comprehensive and Progressive Agreement for Trans-Pacific Partnership (CPTPP) to expand economic collaboration with crucial US partners within the region. Instead of directly competing with China in regions where China already has significant influence, the US may use a strategy of multilateral cooperation with its partners [36]. Fiscal decentralization and empowering the US local governments may have a positive impact on economic performance to design effective policies for the resilience of the US economy [33].

Although most African countries also received a high percentage of FDI from China in 2019 (e.g., Comoros [100%], Djibouti [97%], South Sudan [94%]), they did not play a significant role in creating vulnerability to the US economy, because the US did not import high volumes of commodities from these African countries (Fig 3).

### Commodities that are conduits for indirect US economic vulnerability to China

To identify commodities that potentially induce indirect vulnerability of the US economy to China, we took the average across intermediary countries of each commodity’s PIVIs (Table 3); that is, for commodity *j*, we calculated $\left(1/I\right)\sum_{i=1}^{I}{PIVI}_{i,j}^{\left(\text{China},\text{US}\right)}$, where *I* is the number of intermediary countries with net export of commodity *j* to the US. Our results suggested that commodities that induced the most indirect vulnerability of the US economy in 2019 were a mix of agricultural products, energy-related resources, and critical minerals, with swine (imported solely from Canada) at the top of the list (Table 3). These vulnerability-inducing commodities can vary greatly depending on global demand, market prices, economic conditions, and trade agreements. The interested reader is referred to S1 Table for the top 50 vulnerability-inducing commodities out of 1,220 4-digit products in 2019.

It is worth noting that almost 10% of Cadmium (HS-8107) and commercial Cobalt oxides and hydroxides (HS-2822) came to the US directly from China (direct vulnerability) and 90% from other partner countries that are dependent on China through the inflow of FDI. These commodities have extensive utilization in battery production, electrical conductors, galvanizing and electroplating processes, alloy manufacturing, pigment and plastic fabrication, and stabilization of phosphate fertilizers [44, 45], collectively constituting important sectors of the US economy.

### Sector-specific vulnerability: An example of critical minerals

In addition to the vulnerability of the overall economy, one can use PIVI to determine the indirect vulnerability of a specific sector of interest. As an example, we perform an in-depth examination of minerals identified as critical to US manufacturing by RAND Corporation [32, 46]. These critical minerals are primarily produced in one or a few countries and meet the following criteria: (1) The primary producer(s) is located outside of the US; (2) there is a significant volume of net imports to the US; and (3) the primary producer(s) exhibits deficiencies in quality of governance, as indicated by the Worldwide Governance Indicators. China is the only nation that meets these criteria and produces more than 50% of the World’s production for multiple critical minerals [32]. China exerts significant control over US access to these critical metals, which highlights the potential risks associated with this interdependence. Among these critical minerals, Cobalt ores (HS-2605) had the highest average PIVI across all countries, followed by Tungsten ores (HS-2611) and Copper (HS-7405) in 2019 (Table 4). China has emerged as a central player in the global trade network for cobalt and copper, enhancing its ability to influence international trade relations [47]. Cobalt stands out as a vital resource for the transition from fossil fuels to renewable energy sources [27]. Tungsten has a wide range of applications such as cutting tools, electrical lighting, aerospace and defence applications due to its high density nature, high-temperature mechanical characteristics, and its electrical conductivity; finding substitutes for Tungsten is challenging because it either raises costs or reduces the performance of the products they are used in [32, 48]. Given the important roles these minerals play in the US economy and their high PIVI values, they can potentially pose national security risks.

### Future work and caveats

The PIVI analysis can be naturally expanded in many ways. In this paper, we focused on the 2019 dataset. However, the pattern of vulnerability can change over time due to such factors as economic conditions, geopolitical dynamics, technological advancements, shifts in global trade, and global pandemic. Applying the PIVI analysis to the datasets across different years will reveal how the vulnerability co-evolves with these factors. Similarly, the pandemic has had a significant and far-reaching effect on global trade and economies worldwide. It has disrupted supply chains, caused a decline in global trade, and led to economic recessions in many countries. Policy responses to address the pandemic, such as lockdowns and travel restrictions have also influenced the economic landscape and may have long-term consequences. Thus, it is important to examine the dynamics and the impacts of COVID-19 on the pattern of vulnerability in future work.

We focused on the US and China in this paper, but the PIVI analysis can be readily applied to any target-investing pairs of countries. Expanding the analysis to cover all pairs of countries across different years will result in a global dynamic network of indirect vulnerability induced by economic interdependence, which will greatly contribute to our more comprehensive understanding of such hidden vulnerability.

The aggregation done in the analysis above gave equal weights to all country-commodity pairs. However, it is possible—or even desirable for some purposes—to include additional considerations and/or qualitative factors that would deliver policy makers a more nuanced and contextualized measure of vulnerability. For instance, substitutability of a commodity is not considered here, and a commodity that has many possible substitutes might weigh less than a commodity with the same PIVI that has no substitutes (think Uranium). Other influential factors include national priorities. For example, commodities critical to the functioning of the US military might be considered more important than commodities with the same PIVI that are not critical to national security. While such considerations are beyond the scope of this paper, they offer rich opportunities for future research and extensions of this work.

It is also important to acknowledge that the current version of the index of vulnerability does not incorporate considerations of political relationships between countries. This also presents an opportunity for future research to improve the index’s usefulness and applicability. For example, researchers could modify PIVI by factoring in the political alliances and affiliations between countries. This extension would provide a more nuanced assessment of vulnerability, considering aspects such as whether close allies like Canada, despite having significant foreign direct investment in the United States, actually pose a substantial risk. By incorporating political relationship dynamics, researchers could enrich the index of vulnerability, providing a more comprehensive evaluation of the interplay between economic and political factors. This expanded perspective would yield valuable insights for policymakers and researchers seeking a more nuanced understanding of the indirect vulnerabilities and their implications on a global scale.

## Conclusion

In this study, we have developed a novel index, called Potential Indirect Vulnerability Index (PIVI), to quantify subtle, hidden vulnerabilities that arise in the interconnected trade networks and investment networks. To demonstrate its application, we used PIVI to determine the indirect vulnerabilities to the US economy due to interdependence with China based on 2019 data, which included the most recent pre-pandemic data of trade flows and FDI. Many countries that induced such indirect vulnerability were Asian countries with geographical proximity with China, with Vietnam at the top of the list. The commodities that induced the most indirect vulnerability in 2019 were a mix of agricultural products, energy-related resources, and critical minerals, with swine at the top of the list. Among the minerals identified as critical to US manufacturing security, our results show that cobalt induces the most indirect vulnerability, followed by tungsten and copper. Some potential ways to reduce such indirect vulnerability for the US are to increase diversity in trade partners, promote foreign direct investment in critical nations such as Vietnam, and/or establish trade agreements with ASEAN nations and TPP countries to strengthen the United States’ economic influence in the region.

Finally, it is important to note that the PIVI is a building block that can be extended and modified in many ways, e.g., considering temporal evolution, more pairs of target and investing countries as well as incorporating such factors as national priorities and political relationships; these extensions can offer a more nuanced and comprehensive understanding of the indirect vulnerability at the global scale, thereby informing policymakers about potential security implications of trade and financial policies.

## Supporting information

S1 Table Top 50 commodities out of 1,220 4-digit ones that potentially induce indirect vulnerabilities of the US economy to China in 2019. (DOCX)
