# Supplementary material for: Quantifying economic vulnerabilities induced by interdependent networks
Source: PLoS One. 2024 Jul 11;19(7):e0306893. doi: 10.1371/journal.pone.0306893 (PMC11239037; doi:10.1371/journal.pone.0306893)
Supplement: S1 Table — (DOCX) [file pone.0306893.s001.docx]

Supporting information

**Quantifying economic vulnerabilities induced by interdependent networks**

Shokoufeh Pourshahabi, Shade T. Shutters, Rachata Muneepeerakul

| **Table S1.** Top 50 commodities out of 1,220 4-digit ones that potentially induce indirect vulnerabilities of the US economy to China in 2019. | | | |
| --- | --- | --- | --- |
| **Rank** | **Mean PIVI^*^** | **Commodity code** | **Commodity description** |
| 1 | 2.78 | 0103 | Swine; live |
| 2 | 2.77 | 1203 | Copra |
| 3 | 2.61 | 2716 | Electrical energy |
| 4 | 1.86 | 0205 | Meat; of horses, asses, mules or hinnies, fresh, chilled or frozen |
| 5 | 1.85 | 2705 | Coal gas, water gas, producer gas and similar gases, other than petroleum gases and other gaseous hydrocarbons |
| 6 | 1.85 | 8107 | Cadmium; articles thereof, including waste and scrap |
| 7 | 1.71 | 2605 | Cobalt ores and concentrates |
| 8 | 1.67 | 2822 | Cobalt oxides and hydroxides; commercial cobalt oxides |
| 9 | 1.60 | 5110 | Yarn of coarse animal hair or of horsehair (including gimped horsehair yarn), whether or not put up for retail sale |
| 10 | 1.43 | 2528 | Natural borates and concentrates thereof (whether or not calcined), but not including borates separated from natural brine; natural boric acid containing not more than 85 % of H3BO3 calculated on the dry weight |
| 11 | 1.38 | 3601 | Explosives; propellent powders |
| 12 | 1.33 | 7603 | Aluminium; powders and flakes |
| 13 | 1.31 | 3205 | Colour lakes; preparations based on colour lakes as specified in note 3 to this chapter |
| 14 | 1.30 | 8601 | Rail locomotives; powered from an external source of electricity or by electric accumulators |
| 15 | 1.30 | 2611 | Tungsten ores and concentrates |
| 16 | 1.29 | 2305 | Oil-cake and other solid residues; whether or not ground or in the form of pellets, resulting from the extraction of ground-nut oil |
| 17 | 1.29 | 2820 | Manganese oxides |
| 18 | 1.25 | 4502 | Natural cork, debacked or roughly squared, or in rectangular (including square) blocks, plates, sheets or strip, (including sharp-edged blanks for corks or stoppers) |
| 19 | 1.23 | 5002 | Raw silk (not thrown) |
| 20 | 1.23 | 7504 | Nickel; powders and flakes |
| 21 | 1.18 | 5005 | Yarn spun from silk waste, not put up for retail sale |
| 22 | 1.18 | 7405 | Copper; master alloys |
| 23 | 1.18 | 5104 | Wool, or fine or coarse animal hair; garneted stock |
| 24 | 1.15 | 4702 | Chemical wood pulp, dissolving grades |
| 25 | 1.13 | 2525 | Mica, including splitting’s; mica waste |
| 26 | 1.10 | 0814 | Peel of citrus fruit or melons (including watermelons); fresh, frozen dried or provisionally preserved in brine, in sulphur water or in other preservative solutions |
| 27 | 1.07 | 0508 | Coral and similar materials, unworked or simply prepared, shells of molluscs, crustaceans or echinoderms, not cut to shape powder and waste thereof |
| 28 | 1.07 | 2801 | Fluorine, chlorine, bromine and iodine |
| 29 | 1.06 | 4703 | Chemical wood pulp, soda or sulphate, other than dissolving grades |
| 30 | 1.06 | 2706 | Tar distilled from coal, from lignite, peat and other mineral tars, whether or not dehydrated or partially distilled; including reconstituted tars |
| 31 | 1.05 | 0812 | Fruit and nuts provisionally preserved; e.g. by sulphur dioxide gas, brine, in sulphur water or in other preservative solutions, but unsuitable in that state for immediate consumption |
| 32 | 1.04 | 4014 | Hygienic or pharmaceutical articles (including teats), of vulcanised rubber other than hard rubber, with or without fittings of hard rubber |
| 33 | 1.01 | 7505 | Nickel; bars, rods, profiles and wire |
| 34 | 1.01 | 2823 | Titanium oxides |
| 35 | 1.00 | 5003 | Silk waste (including cocoons unsuitable for reeling, yarn waste and garneted stock) |
| 36 | 1.00 | 8703 | Motor cars and other motor vehicles; principally designed for the transport of persons (other than those of heading no. 8702), including station wagons and racing cars |
| 37 | 0.99 | 7220 | Stainless steel; flat-rolled products of width less than 600mm |
| 38 | 0.99 | 8106 | Bismuth; articles thereof, including waste and scrap |
| 39 | 0.99 | 8709 | Works trucks, self-propelled, (not fitted with lifting or handling equipment), for factories, warehouses etc., for short distance transport of goods, tractors used on railway station platforms; parts thereof |
| 40 | 0.98 | 5306 | Flax yarn |
| 41 | 0.98 | 2513 | Pumice stone; emery; natural corundum, natural garnet and other natural abrasives, whether or not heat treated |
| 42 | 0.98 | 5809 | Fabrics, woven; of metal thread and metallised yarn of heading no. 5605, of a kind used in apparel, as furnishing fabrics or similar purposes; N.E.C. or included |
| 43 | 0.98 | 3906 | Acrylic polymers in primary forms |
| 44 | 0.97 | 9303 | Firearms; other similar devices (e.g. sporting shotguns and rifles, muzzle-loading firearms, very pistols, devices for firing flares or blank ammunition, captive bolt humane killers, line throwing guns) |
| 45 | 0.97 | 2615 | Niobium, tantalum, vanadium or zirconium ores and concentrates |
| 46 | 0.96 | 7223 | Stainless steel wire |
| 47 | 0.95 | 5803 | Gauze; other than narrow fabrics of heading no. 5806 |
| 48 | 0.95 | 1903 | Tapioca and substitutes therefor prepared from starch; in the form of flakes, grains, pearls, siftings or similar forms |
| 49 | 0.95 | 5905 | Textile wall coverings |
| 50 | 0.94 | 0907 | Cloves (whole fruit, cloves and stems) |
| ^*^Here, the mean PIVI of commodity *j* is defined as $1/{I\sum_{i=1}^{I} {PIVI}_{i,j}^{(China,US)}}$ where *I* is the number of intermediary countries with net export of commodity *j* to the US. | | | |
